# Crosstalk between monocytes and myometrial smooth muscle in culture generates synergistic pro-inflammatory cytokine production and enhances myocyte contraction, with effects opposed by progesterone

**DOI:** 10.1093/molehr/gav027

**Published:** 2015-05-22

**Authors:** S.P. Rajagopal, J.L. Hutchinson, D.A. Dorward, A.G. Rossi, J.E. Norman

**Affiliations:** 1 MRC Centre forReproductive Health, University of Edinburgh, Queen's Medical Research Institute, 47 Little France Crescent, Edinburgh EH16 4TJ, UK; 2 MRC Centre for Inflammation Research, University of Edinburgh, Queen's Medical Research Institute, 47 Little France Crescent, Edinburgh EH16 4TJ, UK

**Keywords:** Contraction, inflammation, monocyte, myocyte, preterm labour

## Abstract

Both term and preterm parturition are characterized by an influx of macrophages and neutrophils into the myometrium and cervix, with co-incident increased peripheral blood monocyte activation. Infection and inflammation are strongly implicated in the pathology of preterm labour (PTL), with progesterone considered a promising candidate for its prevention or treatment. In this study, we investigated the effect of monocytes on myometrial smooth muscle cell inflammatory cytokine production both alone and in response to LPS, a TLR4 agonist used to trigger PTL *in vivo*. We also investigated the effect of monocytes on myocyte contraction. Monocytes, isolated from peripheral blood samples from term pregnant women, were cultured alone, or co-cultured with PHM1-41 myometrial smooth muscle cells, for 24 h. In a third set of experiments, PHM1-41 myocytes were cultured for 24 h in isolation. Cytokine secretion was determined by ELISA or multiplex assays. Co-culture of monocytes and myocytes led to synergistic secretion of pro-inflammatory cytokines and chemokines including IL-6, IL-8 and MCP-1, with the secretion being further enhanced by LPS (100 ng/ml). The synergistic secretion of IL-6 and IL-8 from co-cultures was mediated in part by direct cell–cell contact, and by TNF. Conditioned media from co-cultures stimulated contraction of PHM1-41 myocytes, and the effect was inhibited by progesterone. Both progesterone and IL-10 inhibited LPS-stimulated IL-6 and IL-8 secretion from co-cultures, while progesterone also inhibited chemokine secretion. These data suggest that monocytes infiltrating the myometrium at labour participate in crosstalk that potentiates pro-inflammatory cytokine secretion, an effect that is enhanced by LPS, and can augment myocyte contraction. These effects are all partially inhibited by progesterone.

## Introduction

Human term labour is an inflammatory event, characterized by localized pro-inflammatory mediator production, leukocyte infiltration, and tissue remodelling occurring within the uteroplacental unit. The mechanisms controlling labour initiation at term are still to be fully elucidated but involve myometrial stretch, progesterone withdrawal, fetal-derived signals and changes in immune regulators (Voltolini *et al.*, 2013). Preterm labour (PTL, defined as labour before 37 weeks of gestation) is the leading cause of neonatal mortality and morbidity (Lawn *et al.*, 2010). Whilst survival rates have improved in recent years, the rate of preterm birth itself is increasing in most countries (Blencowe *et al.*, 2012). The causes of PTL are multifactorial, and there is a strong association between intrauterine inflammation and infection (Goldenberg *et al.*, 2008), with ∼40% of all PTL attributable (at least in part) to infection/inflammation (Lamont, 2003). This connection is strengthened by evidence that bacterial wall extract lipopolysaccharide (LPS), a TLR4 agonist, initiates PTL in animal models of pregnancy (Elovitz *et al.*, 2003; Adams Waldorf *et al*., 2008).

The myometrium generates powerful synchronized contractions leading to successful delivery. These synchronized contractions in turn are dependent on the individual contractile activity of the uterine myocytes. Contractile activity is controlled by calcium sensitization, myosin phosphorylation and excitation-contraction coupling (Aguilar and Mitchell, 2010). Tocolysis, which targets the inhibition of contractions has been the main treatment approach for women in PTL, albeit with limited efficacy and limited improvement in the neonatal mortality rate (Haas *et al.*, 2012). Given the association with infection/inflammation, more recent approaches to PTL treatment or prevention have shifted to a focus on compounds with combined anti-inflammatory and anti-contractile properties such as progesterone (Rinaldi *et al.*, 2011; Voltolini *et al.*, 2013).

The inflammatory events of labour include activation of circulating leukocytes (Luppi *et al.*, 2004; Yuan *et al.*, 2009) and an influx of macrophages and neutrophils into the myometrium (Thomson *et al.*, 1999; Osman *et al.*, 2003; Hamilton *et al.*, 2012). This influx is coincident with myometrial transformation from a refractory and quiescent organ, into one which contracts in a coordinated manner in response to uterotonics (Monga *et al.*, 1996; Olson, 2003). At term, this leukocyte trafficking is likely regulated in part by stretch-driven chemokine secretion (Hua *et al.*, 2012; Lee *et al.*, 2014). The infiltrating macrophages are recruited from the maternal circulation as monocytes, which begin to differentiate upon extravasation and trafficking from the uterine vasculature (Kim *et al.*, 2007; Leong *et al.*, 2008; Srikhajon *et al.*, 2014).

The role(s) of macrophages in the myometrium at labour are uncertain, although in a mouse model of PTL, macrophage depletion delayed labour induction by LPS, highlighting a potential role for these leukocytes in the progression of parturition (Gonzalez *et al.*, 2011). In other systems, monocyte-smooth muscle cell co-incubation has been used to explore the effects of cell–cell crosstalk on TLR2/ TLR4 signalling and cytokine secretion (Morris *et al.*, 2005). The specific aims of this study were to characterize monocyte-myometrial inflammation both alone and in the presence of LPS, to investigate the effects on inflammation of potential PTL therapies including progesterone on these cell types and to examine the connection between inflammation and myocyte contraction. We used primary monocytes obtained from pregnant women at term, and myometrial smooth muscle cell lines. We hypothesized that monocytes would stimulate production of pro-inflammatory cytokines and contraction of myocytes in co-culture, and that the monocyte-myocyte inflammatory responses would be potentiated by LPS.

## Materials and Methods

### Patient samples and cell lines

Blood samples were obtained through the Edinburgh Reproductive Tissue BioBank from healthy women either undergoing elective Caesarean section (*n* = 21), or induction of labour (*n* = 11) all at term (38–42 weeks of gestation) prior to the onset of labour. Informed and written consent was obtained according to the ethical approval and governance granted to the Edinburgh Reproductive Tissues Biobank by the West of Scotland Research Ethics Committee 4 (09/S0704/3). Reasons for elective Caesarean section were previous section/uterine surgery (*n* = 16), breech presentation (*n* = 2) or placental indication (*n* = 3). The sole indication for induction of labour was a gestational age ≥41 weeks. Exclusion criteria included medical complications such as pre-eclampsia, gestational diabetes/hypertension, fetal abnormalities, cervical cerclage, autoimmune disease, smoking, clinical signs of infection and a BMI less than 18 or greater than 29. Blood samples were also obtained from healthy non-pregnant women with informed and written consent, according to ethical approval granted to the MRC Centre for Inflammation Research from Lothian Research Ethics Committee (08/S1103/38). All blood samples were collected into sodium citrate tubes (Monovette, Sarstedt, Leicester, UK).

Primary mononuclear cells and neutrophils were isolated from peripheral blood samples using dextran sedimentation from erythrocytes, and discontinuous Percoll gradients as described (Haslett *et al.*, 1985; Dorward *et al.*, 2013). Monocytes were subsequently purified from peripheral blood mononuclear cells by negative selective with magnetic beads (Monocyte isolation kit II, Miltenyi Biotec, Bisley, UK). Typical purities for monocyte and neutrophil preparations were >95%, as assessed by cytocentrifuge preparation based on H&E staining, and flow cytometry based on FSC/SSC properties.

The pregnant human myometrial 1-41 (PHM1-41) immortalized cell line was a kind gift from B. Sanborn (Colorado State University), and was derived and characterized as previously described (Burghardt *et al.*, 1996). Primary uterine smooth muscle cells (UtSMCs) were obtained from Lonza (Slough, UK), and were derived from a non-pregnant donor by enzymatic dispersion. PHM1-41 cells and UtSMCs were maintained in high glucose Dulbecco's modified Eagle's medium (Lonza) supplemented with 10% fetal calf serum, penicillin/streptomycin (both PAA Laboratories, Yeovil, UK) and l-glutamine (Sigma, Poole, UK) as previously described (Hutchinson *et al.*, 2014).

### Reagents and equipment

LPS from *Escherichia coli* 0111:B (L3024) and progesterone (P10130) were obtained from Sigma. Rabbit monoclonal anti-human TNF neutralizing antibody (D1B4) was obtained from Cell Signalling Technology (Hitchin, UK). Recombinant human IL-10 (217-IL-005) was obtained from R&D Systems (Minneapolis, MN, USA). All were reconstituted in PBS where appropriate, except for progesterone that was reconstituted in DMSO (Sigma). Transwell inserts with 0.4 µm pore diameter were obtained from Corning (Fisher Scientific, Loughborough, UK).

### Co-culture model

UtSMCs or PHM1-41 cells were seeded 24 h prior to the start of experiments in 6-well plates at 1 × 10^5^ cells and 2 × 10^5^ cells/well respectively, giving a confluence of 70–80% by the experiment start for both cell types. These cells were cultured either alone, with primary monocytes (in a 10:1 ratio with the myocytes: monocytes) or alternatively primary monocytes were cultured alone (1 × 10^4^ or 2 × 10^4^ cells for UtSMCs or PHM1-41 experiments respectively). Cells were treated for 24 h, after which conditioned media was collected and centrifuged at 10 000*g* to remove any cellular debris. The resulting supernatants were either stored at −80°C, or used to treat collagen gels immediately.

### Protein analysis

IL-6 and IL-8 Duoset enzyme linked immunosorbent assays and Versa-MAP™ luminex assays (R&D Systems) were performed according to the manufacturer's instructions. Cytokines examined by VERSA-MAP were IL-1α, IL-1β, IL-1RA, TNF, IL-10, G-CSF, GM-CSF, MCP-1/CCL2, MIP-1α/CCL3, CCL5, CXCL5, IFNγ, VEGF and FGF-basic.

### Contraction assays

Collagen gels with embedded PHM1-41 cells were prepared, using 1 × 10^5^ cells/well in 24-well plates, as previously described (Hutchinson *et al.*, 2014). Gels were equilibrated overnight, and then incubated with conditioned media from either PHM1-41 cells alone or PHM1-41/monocyte co-cultures (in a 10:1 ratio), and subsequently treated with either vehicle (DMSO) or with 10 µM progesterone added into the conditioned media. Cell viability was assessed post-contraction using the CellTitre 96 AqueousOne assay (Promega, Southampton, UK), as previously described.

### Statistical analyses

All experiments were performed a minimum of three times, as indicated in the figure legends, with experimental replicates for each treatment conducted in either triplicate (PHM1-41) or duplicate (UtSMCs), and the mean values from each donor experiment were combined for reporting and analysis. Average data were firstly log transformed to normalize distributions, and then analysed by one- or two-way ANOVA, or *t*-test, using GraphPad Prism (version 6.0, La Jolla, CA, USA) with matched samples tests employed where appropriate. For ANOVA, group-wise comparisons were conducted using Bonferroni, Sidak or Tukey's *post hoc* tests for one- or two-ANOVAs. In order to assess synergism between monocytes and myocytes when cultured in the same well, we additionally compared data from cells cultured together (i.e. in the same well, termed ‘co-culture’), with the sum of data from wells of each cell type cultured separately (termed ‘additive’).

## Results

### Response of myocytes, monocytes and neutrophils to TLR4 agonist LPS

**Figure 1:**
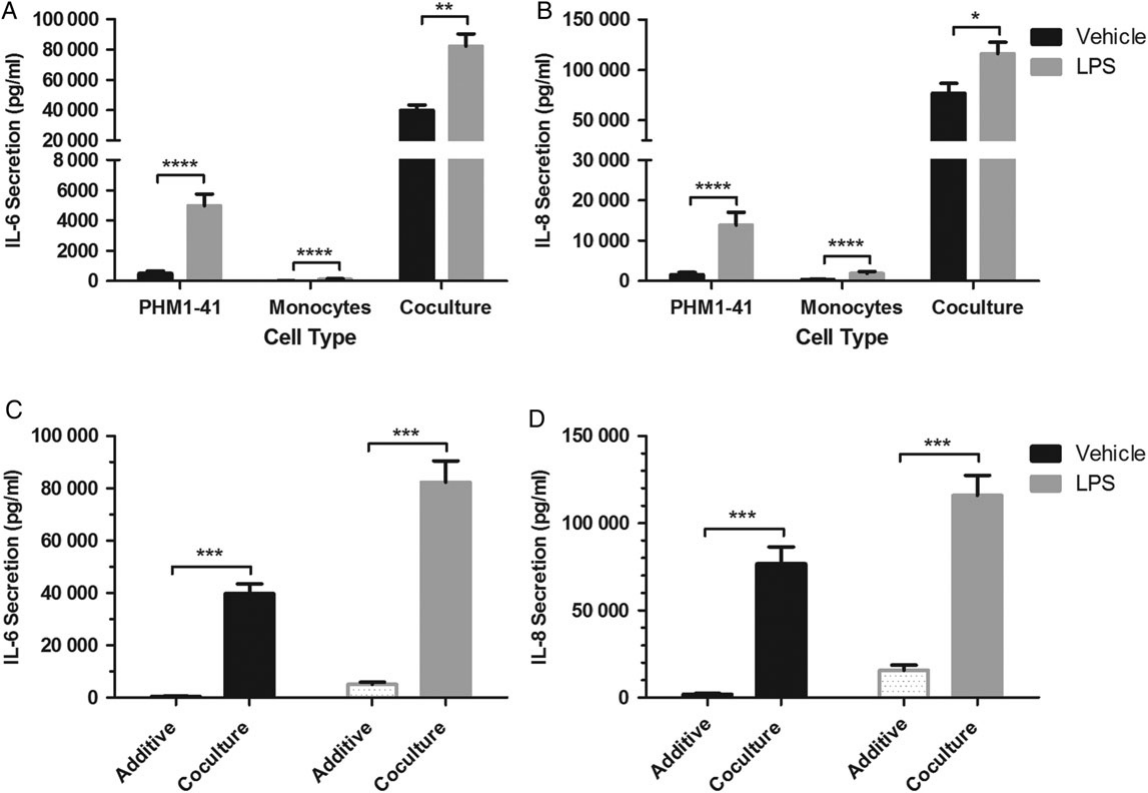
Secretions of IL-6 and IL-8 were enhanced from PHM1-41 myocytes co-cultured with primary monocytes, compared to either cell type cultured separately, either in the presence or absence of the TLR4 agonist LPS. PHM1-41 cells (2 × 10^5^) were cultured alone, or in the presence of primary monocytes from term pregnant women, in a ratio of 10:1, or primary monocytes (2 × 10^4^) were cultured in isolation (*n* = 10). Cells were treated with LPS (100 ng/ml), and supernatants were harvested for ELISA after 24 h and assayed for (**A**) IL-6 and (**B**) IL-8, which were both increased by LPS treatment in each cell combination. Comparison of the additive secretion from PHM1-41 and primary monocytes cultured independently (spotted bars), with PHM1-41/monocyte co-culture (solid bars) for (**C**) IL-6 and (**D**) IL-8 alone, and in the presence of LPS is shown. Note that the ‘additive’ column is the sum of data from experiments with either PHM1-41 cells or primary monocytes cultured separately. Data shown are the mean ± SEM, with each treatment conducted in triplicate and primary monocytes obtained from 10 different donors. Data were analysed by two-way ANOVA and *post hoc* Tukey or Bonferroni multiple comparisons, *P < 0.05, **P < 0.01, ***P < 0.001, ****P < 0.0001.

The effects of LPS treatment on cytokine production from PHM1-41 cells and primary monocytes from term pregnant women was determined, firstly when these cells were cultured separately and secondly in co-culture in a 10:1 ratio. After 24 h of treatment with or without 100 ng/ml LPS, conditioned media concentrations of IL-6 and IL-8 were quantified by ELISA. Levels of both cytokines were significantly increased in response to LPS treatment in each of the wells containing PHM1-41 cells alone (*P* < 0.0001), the wells containing monocytes cultured alone (*P* < 0.0001), and wells with PHM1-41/monocyte co-culture (*P* < 0.01 and *P* < 0.05 for IL-6 and IL-8 respectively, Fig. 1A and B). Notably, the magnitude of both baseline and LPS-induced secretion of IL-6 and IL-8 in the co-cultures was significantly greater than the sum of the values for each cell type cultured separately (*P* < 0.001, Fig. 1C and D). Concentrations in the co-cultures were 90–211 fold higher for LPS-mediated secretion, and 13–221 fold higher for baseline secretion, than the additive data for IL-8 and IL-6 respectively (Fig. 1C and D). These data suggest a role for monocytes participating in crosstalk with myocytes to enhance IL-6 and IL-8 production, both in baseline and in LPS-stimulated conditions.

**Figure 2:**
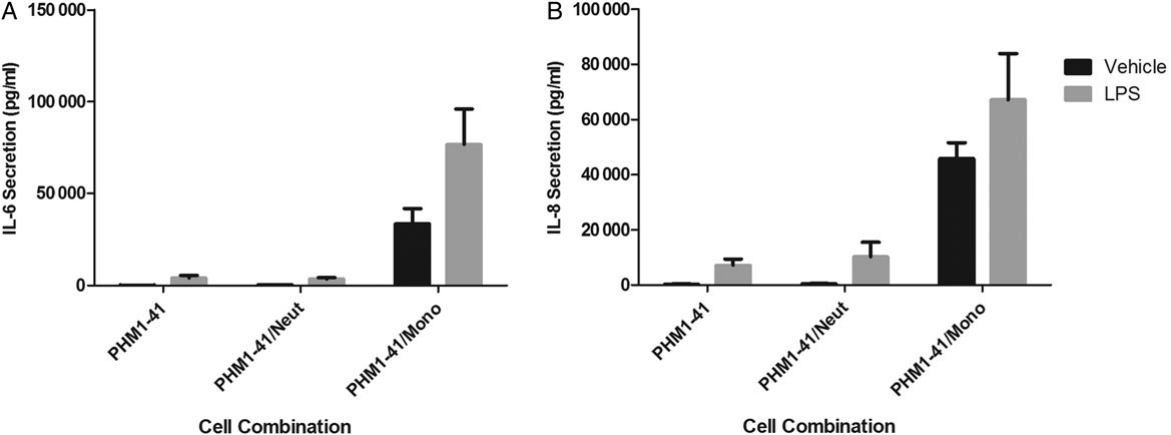
Secretion of IL-6 and IL-8 from neutrophil/PHM1-41 and monocyte/PHM1-41 co-cultures. Supernatants from PHM1-41 cells cultured alone or in a 10:1 ratio with either primary monocytes (Mono) or neutrophils (Neut) from female, non-pregnant donors, in the presence or absence of LPS (100 ng/ml) for 24 h were assayed by ELISA for (**A**) IL-6 and (**B**) IL-8. Data shown are the mean ± SD, *n* = 3 independent experiments conducted in triplicate.

Given that the number and density of neutrophils (as well as macrophages) increases in the myometrium during labour (Thomson *et al.*, 1999), we performed pilot (*n* = 3) experiments to compare the effect of neutrophils with those of monocytes on PHM1-41 cytokine production. PHM1-41 cells were cultured with either primary neutrophils or matched monocytes from the same non-pregnant individuals, at identical concentrations, and treated with or without 100 ng/ml LPS for 24 h, after which IL-6 and IL-8 secretion were determined (Fig. 2A and B). Although the sample size was too small to perform formal statistical analyses, observation of the data did not suggest significant myocyte IL-6 and IL-8 secretion by neutrophils, in contrast to the effect of monocytes. We did not investigate the role of neutrophils further, but decided to focus on the role of monocytes on myometrial inflammation and cytokine secretion in the remainder of the study.

**Figure 3:**
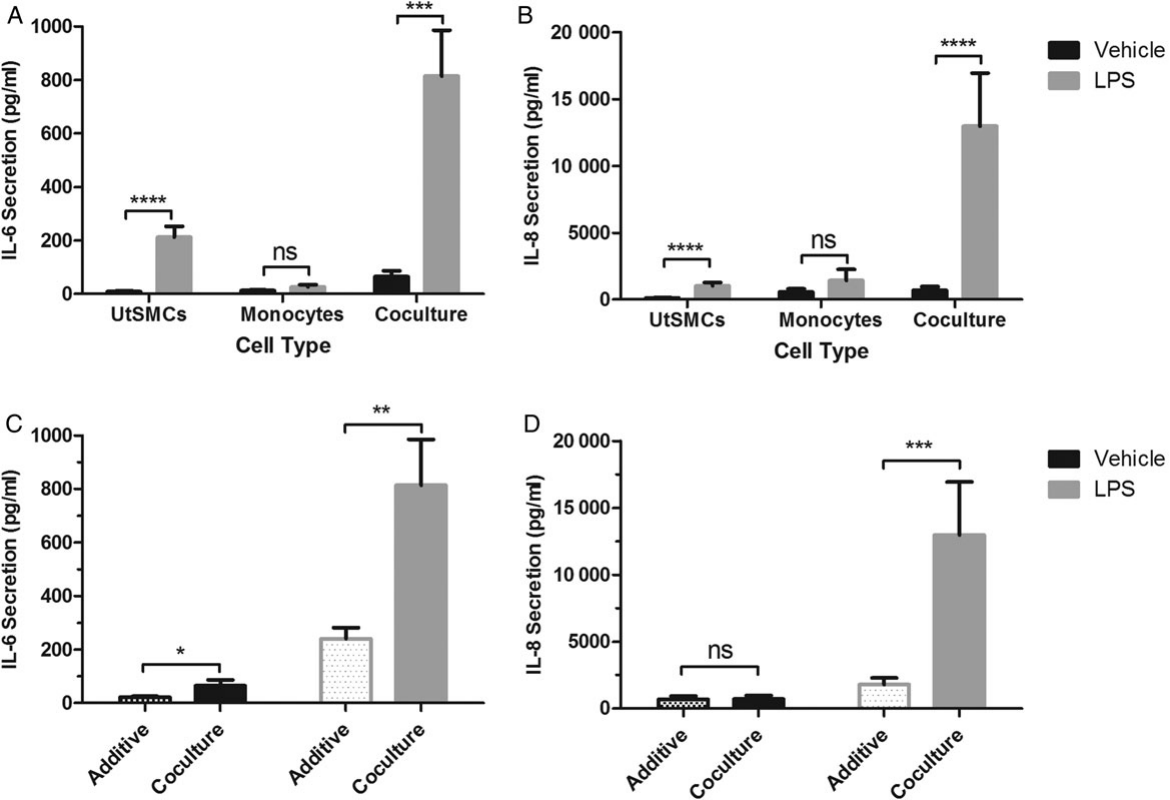
Secretions of IL-6 and IL-8 were enhanced from UtSMCs, in the presence of primary monocytes, compared to either cell type alone, in the presence or absence of LPS. Uterine smooth muscle cells (UtSMCs) were cultured alone (1 × 10^5^ cells), in the presence of primary monocytes from term pregnant women, in a 10:1 ratio, or primary monocytes were cultured in isolation (1 × 10^4^ cells). Cells were treated with 100 ng/ml LPS and supernatants were harvested after 24 h and assayed by ELISA for (**A**) IL-6 and (**B**) IL-8. LPS up-regulated secretion from UtSMCs and UtSMC/monocyte co-culture but not monocytes cultured alone, for both IL-6 and IL-8. Comparison of the additive secretion from UtSMCs and primary monocytes independently cultured (spotted bars), with UtSMC/monocyte co-culture (solid bars) for (**C**) IL-6, found the co-culture had significantly higher secretion of IL-6 both alone and after LPS treatment, whereas for (**D**) IL-8, only after LPS treatment, was the co-culture secretion greater than additive secretion. Data shown are *n* = 5 experiments, and are shown as mean ± SEM. Data were analysed by two-way ANOVA and *post hoc* Tukey or Bonferroni multiple comparisons, *P < 0.05, **P < 0.01, ***P < 0.001, ****P < 0.0001, ns, non-significant.

To examine whether the synergism between myometrial cells and monocytes was restricted to the PHM1-41 cell line, we next examined the behaviour of cultured primary UtSMCs in our co-culture system. As with PHM1-41 cells, IL-6 and IL-8 secretions were enhanced by LPS treatment in the UtSMC/monocyte co-culture (*P* < 0.001 and *P* < 0.0001) and in UtSMCs cultured alone (*P* < 0.0001, Fig. 3A and B). Consistent with the PHM1-41 co-cultures, the magnitudes of LPS-induced secretion of IL-6 and IL-8, were each significantly greater in the UtSMC/monocyte co-cultures than for the additive values from each cell type cultured separately (*P* < 0.01 and *P* < 0.001), as was the case for the baseline secretion of IL-6 (*P* < 0.05, Fig. 3C and D). The magnitude of responses was greatest with LPS treatment, where co-culture secretions of IL-6 and IL-8 were 3-fold and 5-fold greater respectively than the additive values.

### Exploration of cytokine secretion from myocyte/monocyte co-culture

**Figure 4:**
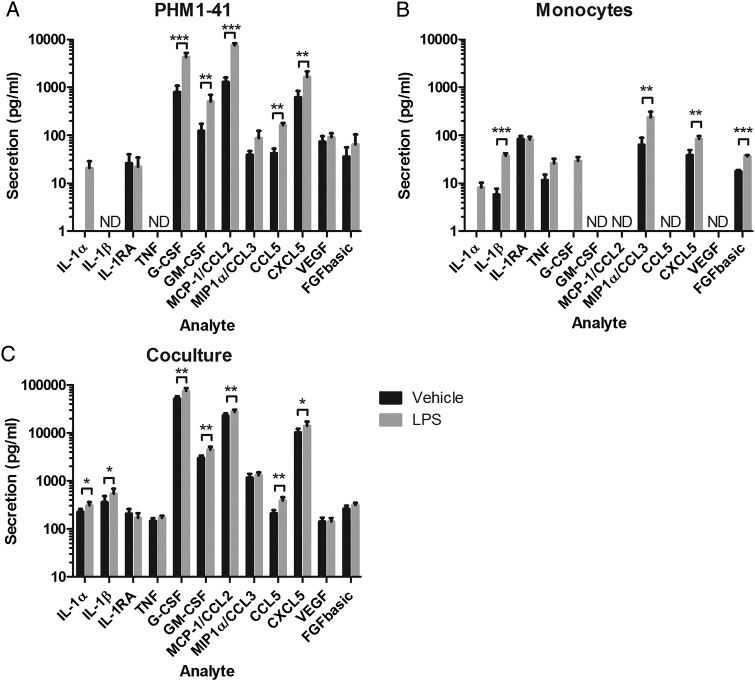
Multiplex assessment of cytokine secretion from vehicle and LPS-treated PHM1-41 cells, primary monocytes and PHM1-41/monocyte co-culture demonstrated differential secretion of multiple factors. PHM1-41 cells were cultured alone, in co-culture with primary monocytes from term pregnant women in a 10:1 ratio, or primary monocytes were culture in isolation, in the presence or absence of 100 ng/ml LPS, treated in triplicate. Supernatants were harvested after 24 h, experiment replicates were pooled and these were assayed for IL-1α, IL-1β, IL-1RA, IL-10, TNF, IFNγ, G-CSF, GM-CSF, MCP-1/CCL2, MIP-1α/CCL3, CCL5, CXCL5, VEGF and FGF-basic using a multiplex system. (**A**) PHM1-41 cells alone, (**B**) primary monocytes alone, and (**C**) PHM1-41/monocyte co-culture. The effects of LPS treatment on the secretion of all cytokines were analysed. Data are shown as the mean ± SEM, obtained from a single multiplex experiment, with samples from eight independent experiments, and were analysed by paired *t*-tests. ND, not detected, *P < 0.05, **P < 0.01, ***P < 0.001.

Further characterization of the myometrial/monocyte co-culture response both alone and after LPS treatment was conducted using a multiplex bead array system to analyse production of a variety of cytokines. Secretion was quantified from the conditioned media of eight independent experiments with pooled replicates, for PHM1-41 cells alone, primary monocytes alone and PHM1-41/monocyte co-cultures, with or without LPS treatment. No secretion of IL-10 or IFNγ was detected from any cell or treatment combination. PHM1-41 cells secreted IL-1RA, G-CSF, GM-CSF, MCP-1/CCL2, MIP-1α/CCL3, CCL5, CXCL5, VEGF and FGF-basic, of which G-CSF, GM-CSF, MCP-1/CCL2, CCL5 and CXCL5 were significantly up-regulated by LPS treatment, and IL-1α secretion was only detected in the presence of LPS (Fig. 4A). Primary monocytes secreted IL-1β, IL-1RA, TNF, MIP-1α/CCL3, CXCL5 and FGF-basic, of which IL-1β, MIP-1α/CCL3, CXCL5 and FGF-basic were up-regulated by LPS treatment, and IL-1α and G-CSF were only detected in the presence of LPS (Fig. 4B). In the co-cultures, all of the aforementioned cytokines were detected, with LPS treatment leading to increased secretion of IL-1α, IL-1β, G-CSF, GM-CSF, MCP-1/CCL2, CCL5 and CXCL5 (Fig. 4C). Again, as for IL-6 and IL-8, the levels of almost all tested cytokines were greater in the co-culture, both alone and after LPS treatment, compared with the additive data for each cell type cultured separately (Table I). Note that additive values were calculated from the independent cultures of PHM1-41 cells and monocytes. Synergism of co-culture secretion was expressed as a ratio of co-culture secretion to additive secretion for both vehicle and LPS treated cells. Comparison of additive versus co-culture secretion of factors, both alone and in response to LPS, showed significantly enhanced secretion of multiple factors. The only exception was the anti-inflammatory cytokine IL-1RA, where there were no significant differences between the additive and the co-culture data. Synergism was greatest for the pro-inflammatory cytokines IL-1β and TNF, and leukocyte growth factors, G-CSF and GM-CSF for vehicle treated co-cultures, and greatest for G-CSF, GM-CSF, IL-1α and IL-1β for LPS treated co-cultures (Table I). Co-culture secretion of chemokines MCP-1/CCL2, MIP-1α/CCL3, CCL5 and CXCL5, and growth factors FGF-basic and VEGF, were also significantly enhanced compared with additive secretion, for both vehicle and LPS treatments suggesting the enhanced production was due to crosstalk between the cell types. Notably, the labour-associated cytokines IL-1β and TNF were only detected in supernatants from primary monocytes and co-cultures, but not from PHM1-41 cells alone.

**Table I:** Synergism of multiple cytokines from myocyte/monocyte co-cultures was detectable in cultures treated alone or with LPS compared with additive secretion of both cell types cultured alone.

	Vehicle				LPS			
Analyte^a^	PHM1-41 + monocytes^b^	Co-culture	Synergism^c^	*P*^d^	PHM1-41 + monocytes^b^	Co-culture	Synergism^c^	*P*^d^
IL-1α	–	228 ± 30	–	–	21 ± 5	308 ± 50	15	***
IL-1β	2.9 ± 1.4	360 ± 124	123	***	37 ± 5	541 ± 141	15	***
IL-1RA	101 ± 15	209 ± 49	2	–	81 ± 13	169 ± 93	2	–
TNF	4.4 ± 2.4	147 ± 20	33	***	26 ± 6	169 ± 20	7	***
G-CSF	803 ± 286	51879 ± 6502	65	***	4301 ± 959	75627 ± 10656	18	***
GM-CSF	62 ± 33	2996 ± 379	48	**	253 ± 130	4495 ± 663	18	**
MCP-1/CCL2	1316 ± 289	22878 ± 2485	17	***	7585 ± 795	27733 ± 3057	4	***
MIP-1α/CCL3	74 ± 32	1160 ± 237	16	**	259 ± 81	1302 ± 198	5	**
CCL5	37 ± 11	211 ± 35	6	***	164 ± 19	389 ± 68	2	***
CXCL5	661 ± 217	10277 ± 2003	16	***	1743 ± 475	14110 ± 3052	8	***
VEGF	56 ± 20	143 ± 28	3	**	69 ± 20	143 ± 25	2	**
FGFb	36 ± 12	261 ± 45	7	***	69 ± 23	315 ± 34	5	***

^a^All secretion values are reported as mean ± SEM, in pg/ml, *n* = 8 samples.

^b^The sum of secretion for independently cultured PHM1-41 cells and primary monocytes.

^c^Synergism was calculated as the ratio of secretion from the co-culture to the sum of independently cultured cell types.

^d^*P*-values were calculated for each analyte using paired *t*-test analysis after log transformation of data. **P < 0.01, ***P < 0.001.

### Factors regulating cytokine secretion from myocyte/monocyte co-cultures

**Figure 5:**
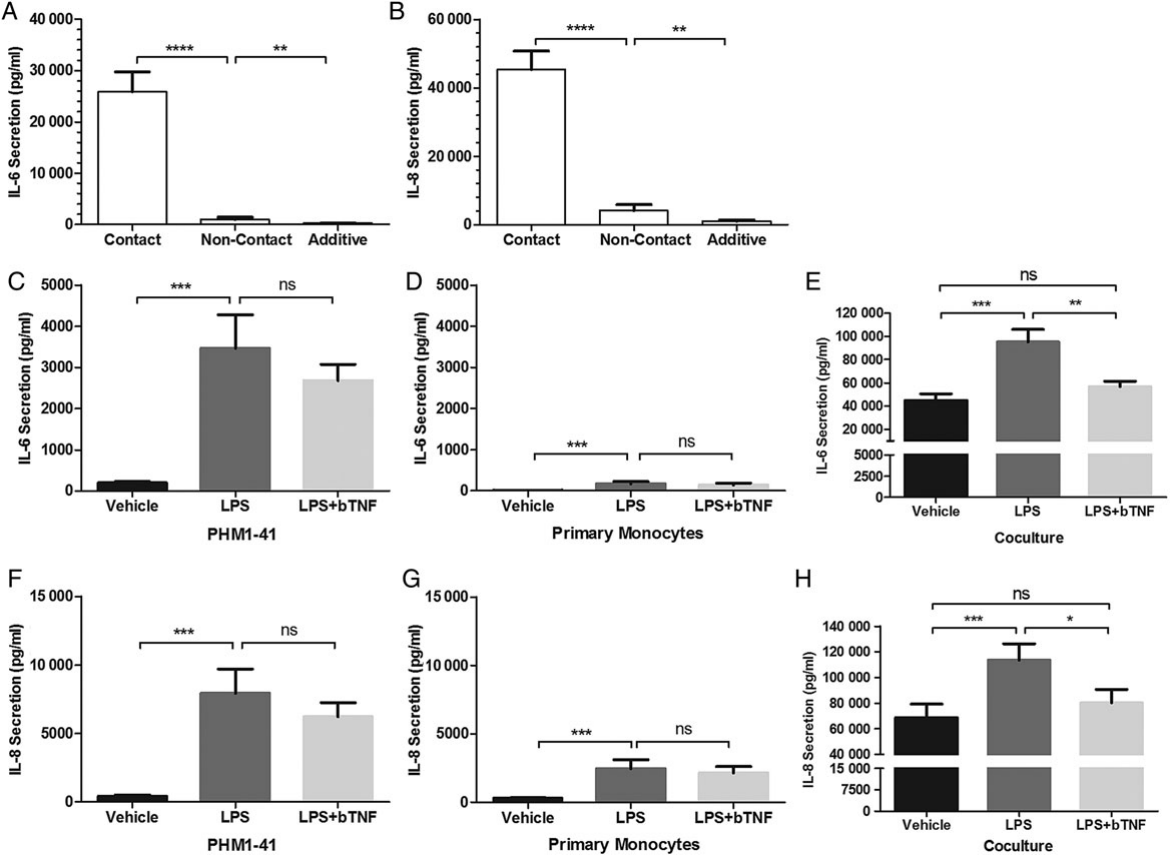
Co-culture secretion of IL-6 and IL-8 were regulated by direct contact and by TNF in LPS-mediated secretion. (**A** and **B**) Both (A) IL-6 and (B) IL-8 secretion from PHM1-41/monocyte co-cultures were enhanced when both cell types were in direct contact, compared to when co-cultured in separate compartments, using transwell inserts (0.4 µM). Cells co-cultured together but not in direct contact had enhanced secretion compared with the additive secretion of both cell types cultured independently, as measured by ELISA. Data shown are the mean ± SEM, and *n* = 8 independent experiments, conducted in duplicate. (**C–H**) Inhibition of TNF signalling using a blocking antibody (bTNF, 50 ng/ml) alongside LPS treatment for PHM1-41 cells alone, PHM1/monocyte co-culture and monocytes alone. Blockade of TNF led to a reduction in LPS-induced IL-6 and IL-8 in the co-culture (E and H), but not for PHM1-41 cells (C and F) or primary monocytes (D and G) treated alone. Data shown are the mean ± SEM, and *n* = 5 independent experiments, conducted in triplicate. Data were analysed by either one-way or two-way ANOVA and *post hoc* Tukey or Sidak multiple comparisons, *P < 0.05, **P < 0.01, ***P < 0.001, ****P < 0.0001, ns, non-significant.

Given the increased cytokine secretion from PHM1-41/monocyte co-cultures (compared to either cell types cultured separately), potential factors that may have influenced this effect were explored. Firstly, the effect of myocyte-monocyte contact on cytokine secretion in an LPS-free environment using a Transwell system was investigated. IL-6 and IL-8 secretion from PHM1-41/monocyte co-cultures were significantly lower when the monocytes had only indirect contact with PHM1-41 cells (*P* < 0.0001, Fig. 5A and B) compared to when cells were in direct contact. Secretions were 25-fold and 11-fold higher from both cell types in direct contact compared with indirect co-cultures for IL-6 and IL-8 respectively. However, indirect (non-contact) co-cultures still had greater than additive secretion from independently cultured cells by 5-fold and 4-fold for IL-6 and IL-8 respectively (*P* < 0.01). These data suggest that both physical contact and secreted factors were critical factors in the observed synergistic pro-inflammatory cytokine response.

Since TNF is up-regulated in the amniotic fluid of women in PTL, as well as in animal models of PTL (Hillier *et al.*, 1993; Hirsch *et al.*, 2006), this cytokine was explored as a potential candidate for the induction of cytokine up-regulation in LPS-treated co-cultures. A blocking antibody was used to inhibit any effects of TNF secretion. The LPS-induced up-regulation in each of IL-6 and IL-8 secretion from either PHM1-41 cells or primary monocytes cultured alone was not significantly lowered by co-treatment with TNF blocking antibody (Fig. 5C, D, F and G). In contrast, in PHM1-41/monocyte co-cultures, LPS-induced IL-6 and IL-8 secretions were significantly suppressed by co-treatment with TNF blocking antibody (*P* < 0.01 and *P* < 0.05), to levels not significantly different from those of vehicle (Fig. 5E and H).

### Functional effects of monocyte-myocyte co-culture and modulators of cytokine secretions in the co-culture

**Figure 6:**
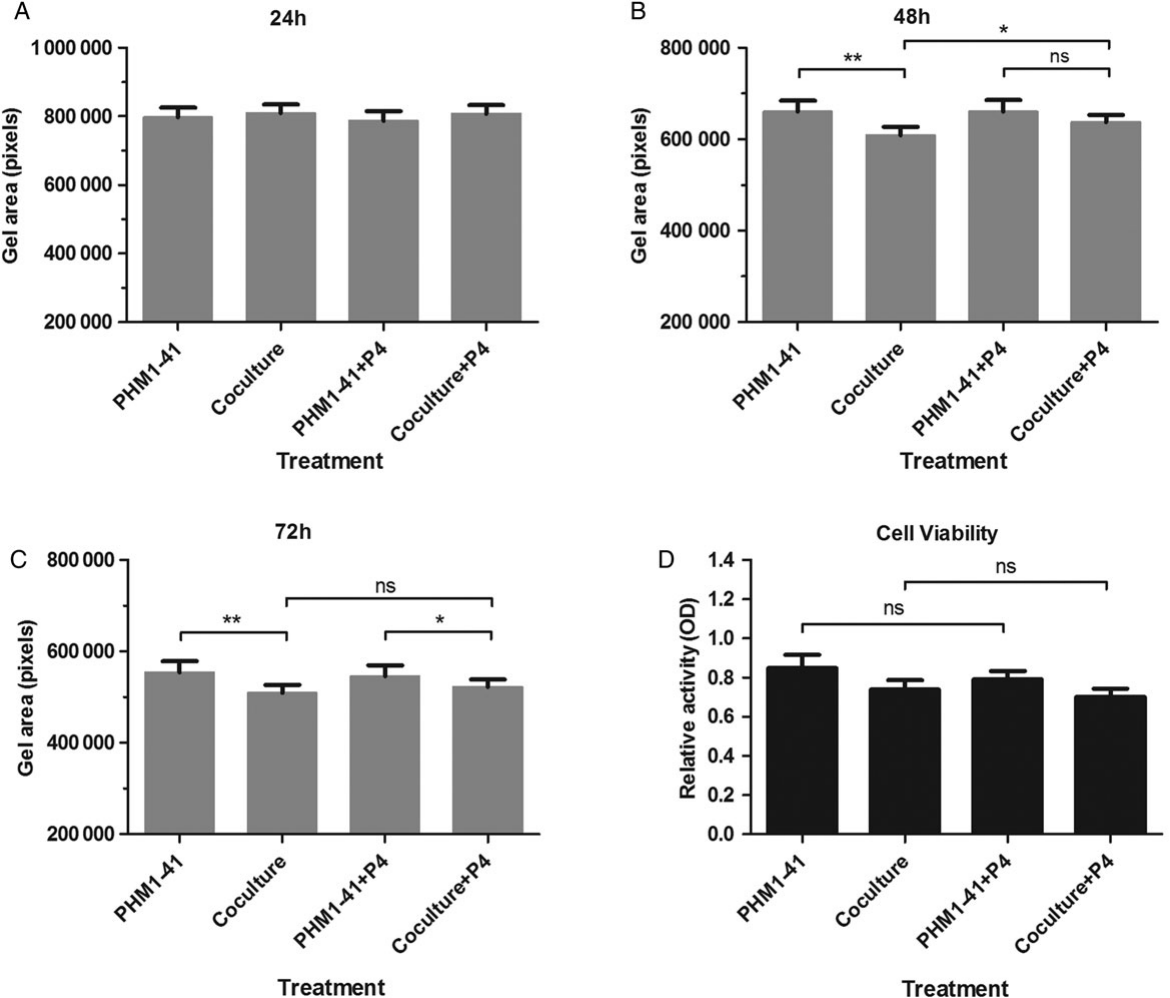
Co-culture-conditioned media enhanced contraction of PHM1-41 cells seeded in collagen gels, with progesterone-mediated inhibition. Collagen gels were embedded with PHM1-41 cells, and treated with conditioned media obtained from either PHM1-41 cells alone or co-cultured PHM1-41/primary monocytes in a 10:1 ratio that were incubated for 24 h. Gel area was monitored over time. (**A**) At 24 h, there was no effect of co-culture or progesterone (P4, 10 µM) treatment on PHM1-41 myocyte contraction. (**B**) At 48 h, conditioned media from co-cultures caused a decrease in gel area (increased contraction) compared with that from PHM1-41 cells alone, and co-culture alone treated gels had a smaller gel area compared with co-culture gels treated with progesterone. (**C**) At 72 h, significantly decreased gel area occurred in both co-culture alone and progesterone treated gels, compared with the treatment controls, with no differences between vehicle and progesterone treatments. (**D**) PHM1-41 gel viable cell numbers was compared between treatment groups using a colourimetric assay, with no differences between any of the treatments. Data are shown as mean ± SEM, *n* = 5 independent experiments conducted with six replicates per treatment. Data were analysed by two-way ANOVA and *post hoc* Sidak or Bonferroni multiple comparisons, ns, non-significant, *P < 0.05, **P < 0.01.

The functional effects of the co-culture on the myocyte itself were also determined, by examining its potential effects on contraction. Conditioned media from PHM1-41 cells cultured either alone, or with primary monocytes in a 10:1 ratio for 24 h, were added to PHM1-41 cells seeded into collagen gel lattices, using an experimental system that we have previously described (Hutchinson *et al.*, 2014). In parallel experiments, either 10 µM progesterone or vehicle (DMSO) was added to the conditioned media, as progesterone has well-established anti-contractile properties (Zakar and Mesiano, 2011). The area of the collagen embedded PHM1-41 cells was monitored over time. There were no significant differences in gel area for any of the treatments after 24 h treatment (Fig. 6A). After 48 h treatment, conditioned media from PHM1-41-monocyte co-cultures significantly enhanced gel contraction (evidenced by smaller gel area) compared with media from PHM1-41 cultures alone (*P* < 0.01, Fig. 6B). Also at 48 h, the enhancement of gel contraction by monocyte PHM1-41 co-cultures was reduced in the presence of progesterone (co-culture versus co-culture + progesterone, *P* < 0.05), and the contraction of progesterone treated gels was not significantly different for PHM1-41 compared with co-culture-conditioned media (Fig. 6B). At 72 h, gels treated with PHM1-41/monocyte conditioned media had enhanced gel contraction compared with the respective controls both in the absence and presence of progesterone (*P* < 0.01 and *P* < 0.05, Fig. 6C), with progesterone no longer inhibiting contraction in co-culture treated cells. Together, these data suggest a potential role for monocyte-myocyte crosstalk not only to alter secretion of cytokines and chemokines, but to enhance myocyte contractile properties, with these effects partially inhibited by progesterone in a time-dependent manner. Importantly, this inhibitory effect of progesterone on myocyte contraction was not a consequence of alterations in cell viability (Fig. 6D).

**Figure 7:**
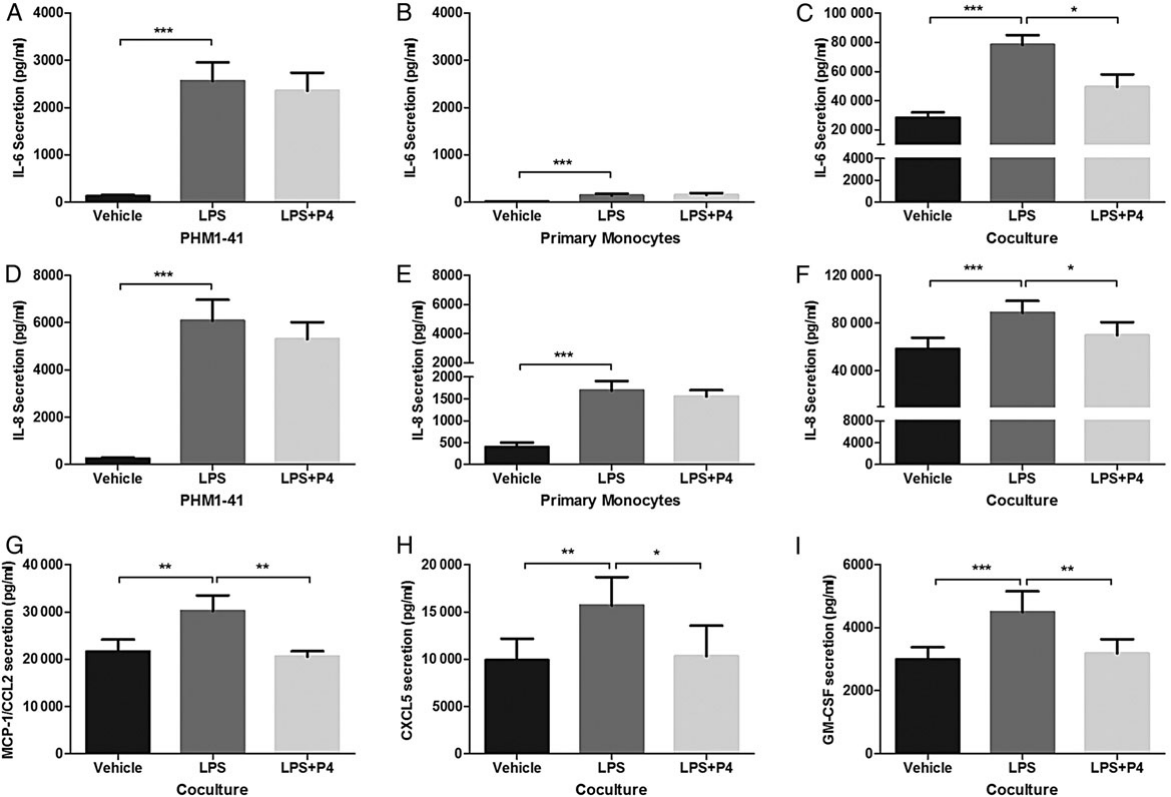
Progesterone inhibited LPS-induced cytokine secretion from PHM1-41/monocyte co-culture, but not from PHM1-41 cells or primary monocytes alone. PHM1-41 cells were cultured alone, in co-culture with primary monocytes from term pregnant women, in a 10:1 ratio, or primary monocytes were cultured alone, and treated with either LPS (100 ng/ml) or LPS and progesterone (P4, 10 µM) for 24 h, after which conditioned media was assayed by ELISA or multiplex assay. IL-6 secretion from (**A**) PHM1-41 cells and (**B**) primary monocytes, were increased by LPS treatment but co-treatment with progesterone had no effect. (**C**) IL-6 secretion from PHM1-41/monocyte co-culture was increased by LPS, and reduced by progesterone co-treatment. IL-8 secretion from (**D**) PHM1-41 cells and (**E**) primary monocytes was increased by LPS treatment, with no effect of progesterone co-treatment. (**F**) IL-8 secretion from the co-culture was increased by LPS, and inhibited by progesterone. (**G**) CCL2 secretion from the co-culture, (**H**) CXCL5 secretion from the co-culture and (**I**) GM-CSF secretion from the co-culture, where LPS treatment led to increased secretion for each, which was inhibited by progesterone co-treatment for each. Data are shown as mean ± SEM, and *n* = 8 independent experiments, conducted in triplicate. Data were analysed by one-way ANOVA and *post hoc* Tukey's multiple comparison, *P < 0.05, **P < 0.01, ***P < 0.001.

Since progesterone inhibited basal and conditioned media myocyte contraction *in vitro* (Fig. 6), its effects on cytokine secretion in the co-culture model was determined. Progesterone failed to inhibit the significant up-regulation of IL-6 and IL-8 secretion that occurred when PHM1-41 cells and primary monocytes alone were each treated with LPS (Fig. 7A, B, D and E). However in the co-cultures, there was significant inhibition of LPS-induced IL-6 and IL-8 secretion by progesterone (*P* < 0.05, Fig. 7C and F). Examination of other secreted factors in the co-cultures found significant suppression by progesterone of LPS-stimulated chemokines/growth factors MCP-1/CCL2 (*P* < 0.01), CXCL5 (*P* < 0.05) and GM-CSF (*P* < 0.01) (Fig. 7G–I). There was no effect of progesterone co-treatment on LPS-induced co-culture secretion of other factors including IL-1β, G-CSF, and notably TNF, given its role in the observed co-culture synergism (data not shown). Together these data demonstrate the specific inhibitory effects of progesterone on LPS-mediated secretion of a number of pro-inflammatory and pro-chemotactic cytokines.

**Figure 8:**
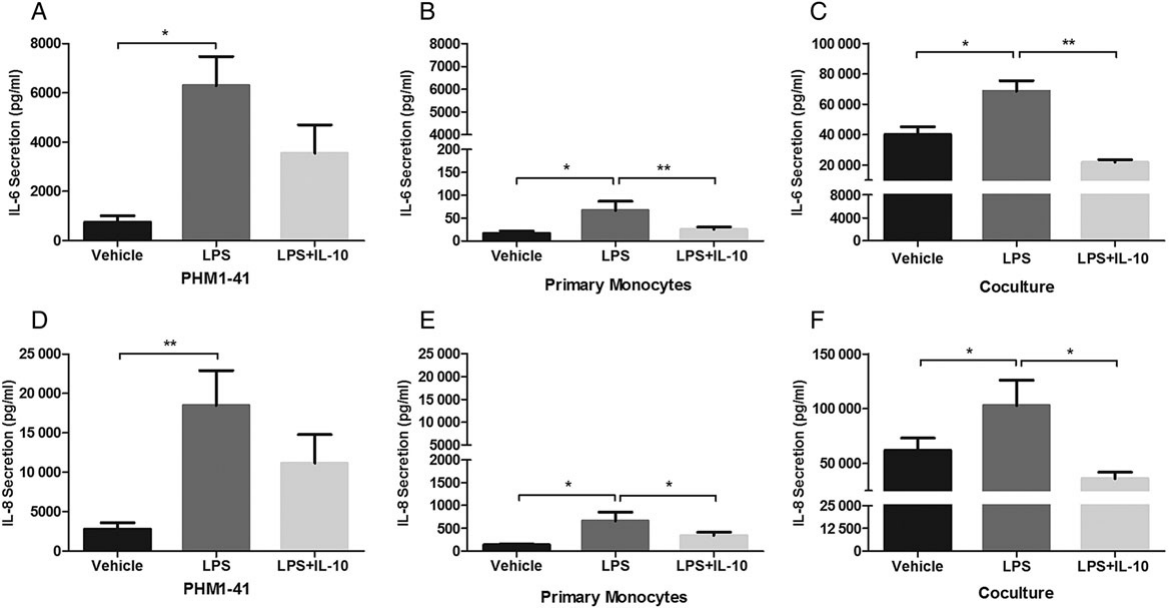
IL-10 inhibited LPS-induced IL-6 and IL-8 secretion from PHM1-41/monocyte co-cultures and primary monocytes, but not from PHM1-41 cells alone. PHM1-41 cells were cultured alone, in co-culture with primary monocytes, in a 10:1 ratio, or primary monocytes were cultured alone, and treated with either LPS (100 ng/ml) or LPS and IL-10 (50 ng/ml) for 24 h, after which conditioned media was assayed by ELISA. (**A**) IL-6 secretion from PHM1-41 cells was increased by LPS, but not inhibited by co-treatment with IL-10. (**B**) IL-6 secretion from primary monocytes was increased by LPS, and inhibited by IL-10. (**C**) IL-6 secretion from PHM1-41/monocyte co-culture was increased by LPS, and inhibited by IL-10. A similar pattern was observed with IL-8, for (**D**) PHM1-41 cells alone, (**E**) primary monocytes alone and (**F**) co-culture. Data are shown as mean ± SEM, *n* = 5 independent experiments, conducted in triplicate. Data were analysed by one-way ANOVA and *post hoc* Tukey's multiple comparison, *P < 0.05, **P < 0.01.

Finally, the classical anti-inflammatory cytokine IL-10 in this model was investigated. Use of this cytokine has been proposed as a treatment for PTL (Robertson *et al.*, 2006; Bayraktar *et al.*, 2009), and it is increased in amniotic fluid of women in PTL (Gotsch *et al.*, 2008). Whilst IL-10 co-treatment had no significant effect on LPS-induced IL-6 and IL-8 secretion from PHM1-41 cells (Fig. 8A and D), the IL-6 and IL-8 releases in both LPS treated primary monocytes (*P* < 0.01 and *P* < 0.05, Fig. 8B and E) and PHM1-41/monocyte co-cultures were inhibited by IL-10 (*P* < 0.01 and *P* < 0.05, Fig. 8C and F). These data suggest that IL-10 influences cytokine release in the co-cultures through the action of monocytes.

## Discussion

**Figure 9:**
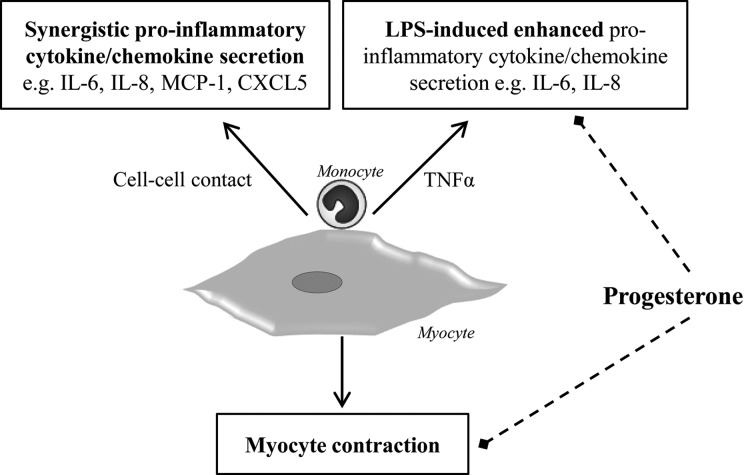
Summary schematic outlining monocyte-myocyte co-culture effects. Coincubation of primary monocytes with myocytes leads to synergistic pro-inflammatory cytokine and chemokine secretion, both alone and in the presence of LPS, and enhanced myocyte contraction (solid arrows). Progesterone (dashed arrows) has an inhibitory effect on both myocyte contraction and pro-inflammatory factor secretion in the presence of LPS.

Myometrial macrophage infiltration at labour has been implicated in both the initiation/propagation of inflammation during parturition as well as the resolution of inflammation and tissue repair post-partum (Hutchinson *et al.*, 2011). Recent studies suggest that macrophage infiltration is an early event occurring at or prior to parturition and that it is regulated by increased chemokine production (Hamilton *et al.*, 2012, 2013; Shynlova *et al.*, 2013b). In this study, we demonstrated the potentiation of pro-inflammatory cytokine secretion from (myometrial) myocyte-monocyte interactions both alone and in response to the bacterial endotoxin, LPS. We also showed that myocyte-monocyte crosstalk could enhance myocyte contraction. From these findings, we propose that monocytes infiltrate into the myometrium at labour (then differentiate into macrophages) and have an enhancing effect on inflammatory mediator production that drives positive feedback loops to further increase pro-inflammatory signals, recruit leukocytes and importantly, alter myocyte contractile machinery to promote myocyte contraction. These may occur in both term and preterm labour. In the presence of infection, the resulting local inflammatory response could recruit monocytes to the myometrium, which then becomes activated and prematurely primed for contraction. These findings provide a novel insight into the possible *in vivo* mechanisms of both term and preterm labour, and support the use of progesterone as an anti-contractile and anti-inflammatory agent, with potential for (partially) inhibiting myometrial inflammation. A summary schematic showing monocyte-myocyte co-culture effects, as evidenced by this study, is shown in Fig. 9.

Both the PHM1-41 and UtSMC cell lines responded to co-culture with monocytes with synergistic secretion of IL-6 and IL-8. In the scenarios of term labour or PTL in the absence of infection, these data suggest that infiltrating monocytes enhance local cytokine secretion, a response which is consistent with reports of enhanced cytokine secretion from myometrial explants taken from women in labour (Young *et al.*, 2002). A recent co-culture study that used the THP-1 monocytic cell line, also found a modest increase in IL-1β and IL-6 gene expression caused by myocyte/THP-1 co-incubation (Thota *et al.*, 2014). However, a contrasting study found no effect of myocyte/THP-1 co-culture on basal cytokine secretion (You *et al.*, 2014). These differing reports could be attributed to disparities in the monocytes used (primary monocytes from term pregnant women versus the THP-1 cell line) or the ratio of cell types used; indeed we also found that THP-1/PHM1-41 co-culture did not alter IL-6 and IL-8 secretion (unpublished observations). The observed synergy in cytokine production was further enhanced by LPS. Hence if infection (e.g. by gram-negative bacteria) became present within the uteroplacental unit triggering chemokine production and consequently monocyte recruitment, then the subsequent myometrial cytokine response would be enhanced. TLR4 signalling in one or both cell types through effector proteins MyD88 and/or TRIF, and subsequent crosstalk between these immune cells and myocytes may be sufficient to trigger a wide-ranging activation of inflammation in both cell types (Patni *et al.*, 2007). Together these data suggest that the synergistic cytokine secretion could be attributed to the monocytes acting on myocytes, to myocytes directing cytokine secretion from the monocytes, or to the mutual communication occurring between both cell types which results in overall enhanced inflammatory mediator secretion.

Our initial study examining cytokine secretion from neutrophil-myocyte co-culture indicated a limited influence of these cell types on IL-6 and IL-8 secretion. Whilst neutrophil recruitment and influx could be important in the process of parturition (e.g. through production of matrix metalloproteinases, reactive oxygen species)(Lee *et al.*, 2014), we have also recently reported that neutrophil ablation does not affect time to delivery in a mouse model of LPS-induced PTL (Rinaldi *et al.*, 2014); these data support the assertion that neutrophil invasion alone may not be critical to parturition.

Physical contact between the myocytes and monocytes was a critical factor in the molecular basis of cooperative synergistic IL-6 and IL-8 response from the co-culture, and had a greater influence on IL-6/IL-8 release than secreted factors alone. Additionally, the pro-inflammatory cytokines IL-1β and TNF, were only secreted by the primary monocytes and in the co-culture (but not from myocytes alone), and therefore are likely to be pivotal in the synergistic cytokine and chemokine secretion from the co-culture. This conclusion was in part supported by TNF blockade, which reduced LPS-induced IL-6 and IL-8 secretion from the co-culture, but not from either cell types cultured independently. An IL-1β/TNF receptor double knockout mouse model had delayed PTL after intrauterine administration of heat-killed *E. coli* (Hirsch *et al.*, 2006), and in a primate model of pregnancy, administration of these cytokines led to induction of labour (Sadowsky *et al.*, 2006), which supports the findings of our study. This research adds to the evidence that IL-1β and TNF are important in the co-culture inflammatory response, and most likely increased by infiltrating leukocytes in the myometrium during labour. Whether infiltrating monocytes themselves are initiators of labour alone *in vivo* is still not clear, but this evidence strengthens the concept of monocytes initiating and potentiating myometrial inflammation.

Monocyte-myocyte co-incubation enhanced the secretion of multiple chemokines including those tropic for monocytes (MCP-1/CCL2, MIP-1α/CCL3, CCL5) and neutrophils (IL-8, CXCL5), and which were enhanced by LPS. These results correlate well with reported chemokine expression and secretion from choriodecidual samples from patients at term and preterm, both labouring and non-labouring (Hamilton *et al.*, 2013). Enhanced myometrial chemokine levels caused by monocyte/myocyte crosstalk at labour would likely regulate activation and extravasation of peripheral leukocytes, from local blood vessels into the uteroplacental unit, in a manner of positive feedback driving inflammation, and therefore labour, forward. These interactions could also be influenced by other sources of pro-inflammatory factors such as the neighbouring decidua, amnion, chorion and placenta (Bollopragada *et al.*, 2009). Use of a broad-spectrum chemokine inhibitor was found to significantly delay LPS-stimulated PTL in a murine model, further highlighting the importance of leukocyte recruitment in labour progression (Shynlova *et al.*, 2014).

At labour, the shift in myocyte physiology from a quiescent to activated and contractile state occurs with increased expression of contractile associated proteins (CAPs) such as connexin-43 and COX-2 (Shynlova *et al.*, 2013a). Since we established that co-culture-conditioned media contained a diverse pro-inflammatory milieu of cytokines and chemokines, we set out to determine if monocytes could affect myocyte contraction. We employed a collagen-based contraction assay, with which we had previously found both rapid and delayed pro-contractile effects on uterine myocytes, of oxytocin and LPS respectively (Hutchinson *et al.*, 2014). In the study described here, a myocyte contractile response stimulated by co-culture-conditioned media was evident after 48 h of treatment, suggesting activation of pathways downstream of cytokine secretion or perhaps accumulation of pro-contractile factors over time. One possible mechanism of enhanced myocyte contraction could involve cytokine-mediated prostaglandin secretion (Rauk and Chiao, 2000; Astle *et al.*, 2007). Additionally, IL-1β and TNF are two likely candidates for the observed myocyte contraction, since they can modulate myocyte intracellular calcium thus enhancing excitability, and directly cause contraction (Tribe *et al.*, 2003; Fitzgibbon *et al.*, 2009; Hutchinson *et al.*, 2014). Recent gene expression data also supports these findings, where monocyte-myocyte co-culture up-regulated CAP expression (Thota *et al.*, 2014). We speculate that these cytokines (and potentially prostaglandins) regulate CAP proteins, thereby priming myocytes for contraction *in vivo*. Further investigation is needed to establish what components of the co-culture-conditioned media caused contraction, and the mechanism by which progesterone inhibited this response.

Both progesterone and IL-10 have been considered for inhibition of PTL (Sadowsky *et al.*, 2003), with progesterone now becoming widely used for prevention (Iams, 2014). Our findings suggest that progesterone (and IL-10) may function at least in part by modulating monocyte-myocyte interactions. Progesterone had wide-ranging effects: both the suppression of LPS-induced cytokines and chemokines (IL-6, IL-8, GM-CSF, CXCL5, MCP-1/CCL2) from the co-culture, and relaxant effects on co-culture-conditioned media induced contraction of myocytes, likely through a number of well-established mechanisms (Allport *et al.*, 2001; Hardy *et al.*, 2006; Jones *et al.*, 2008; Zakar and Mesiano, 2011; Sun *et al.*, 2012). Signalling through myocyte nuclear progesterone receptors (PR) in the PHM1 cells could be responsible for these effects, since PR mRNA expression was detected in our studies (unpublished observations), and in those of others (Madsen *et al.*, 2004; Ciamela *et al.*, 2008). Monocytes are not thought to express PR but do express glucocorticoid receptors (Schust *et al.*, 1996), at which progesterone has weak agonistic activity (Kontula *et al.*, 1983; Svec *et al.*, 1989). The temporal inhibition of co-culture-induced myocyte contraction by progesterone that was evident at 48 h, but not at 72 h, may have been due to progesterone metabolisation over time, an effect which could be further explored by replenishment studies. IL-10 had suppressive effects on LPS-induced IL-6 and IL-8 secretion, which were likely monocyte-mediated in the co-culture given the specificity of inhibition for monocyte and co-culture secretion, but not PHM1-41 cells. Both monocyte expression of IL-10 receptor and the suppression of cytokine secretion from LPS-treated monocytes by IL-10 are well established (De Waal Malefyt *et al*., 1991; Moore *et al.*, 2001). These results suggest monocytes are the principal cell type susceptible to this treatment, and that targeting the monocytes was sufficient to at least dampen LPS-mediated co-culture secretion of IL-6 and IL-8. Future investigation of the manner of action of progesterone and IL-10, including their target cell type(s), would be highly valuable in identifying the mechanisms of pro-inflammatory cytokine inhibition. Together, these data highlight the influence of specific cell types in PTL which could be exacerbating or propagating pro-inflammatory effects, and confirm previously reported progesterone-mediated responses on inflammation and contraction.

Importantly, monocytes are recruited from the peripheral circulation and migrate through the endothelium into tissues, in response to chemokine gradients, and are involved in both the initial and clearance stages of an inflammatory response. In our model, naïve monocytes isolated from peripheral blood, which themselves are a heterogeneous population, were co-cultured with myocytes (Gordon and Taylor, 2005). Refinement of these methods could examine the monocyte subtype(s) responsible for cytokine secretion in response to the myocytes. After 24 h of incubation, in the co-culture, we predict monocyte differentiation into macrophages would have been initiated (although likely not completed), through contact with the myocytes and the influence of growth factors/cytokines such as GM-CSF within the co-culture microenvironment (Iqbal *et al.*, 2014). Characterization of these leukocytes after co-culture, or alternatively differentiation of monocytes to macrophages which are then co-cultured with myocytes, could help to shed light on these questions.

In summary, this study demonstrates for the first time that co-incubation of monocytes and myocytes induces an increased pro-inflammatory response, which is further enhanced by LPS. Co-incubation also stimulates myocyte contraction. This work suggests that at labour, infiltrating macrophages increase local myometrial inflammation through pro-inflammatory cytokines, increased leukocyte trafficking to the myometrium, and enhancement of myocyte contraction. Furthermore, progesterone has multiple effects on monocyte-myocyte interactions, including inhibition of both myocyte contraction and LPS-induced pro-inflammatory factor secretion, further supporting its use in the treatment of PTL. Development of this model using macrophages to recapitulate *in vitro* the macrophage-myocyte interactions that occur in term and preterm labour, could help to extend the understanding of this aspect of myometrial physiology at labour. If the connections between inflammation and contraction in the myometrium, especially the role of monocytes and macrophages in these processes, are better understood, this could aid the search for effective treatments for PTL.

## Authors' roles

S.P.R., J.L.H. and D.A.D. performed the experiments. S.P.R. wrote the manuscript. S.P.R., J.L.H., D.A.D., A.G.R. and J.E.N. contributed to the design of the study, analysis and interpretation of the data, drafting of the article and final approval of the version to be published.

## Funding

This work was supported by the Medical Research Council (Ph.D. studentship to S.P.R, Career Development Fellowship to J.L.H., grants G0601481 and MR/K013386/1 to A.G.R. and grant MR/L002647/1 to J.E.N.), the Wellcome Trust (grant WT096497 to D.A.D.), Action Medical Research and Tommy's the Baby Charity (J.E.N). Funding to pay the Open Access publication charges for this article was provided by the Medical Research Council.

## Conflict of interest

J.E.N. is in receipt of grants from governmental bodies and charities for research projects to understand the pathophysiology of parturition and to treat preterm birth. She is on a data monitoring committee for studies testing interventions to prevent preterm birth for GSK. The other authors declare no conflict of interest.
